# Impact of SGLT2 Inhibitor Therapy on Right Ventricular Function in Patients with Heart Failure and Reduced Ejection Fraction

**DOI:** 10.3390/jcm12010042

**Published:** 2022-12-21

**Authors:** Ivona Mustapic, Darija Bakovic, Zora Susilovic Grabovac, Josip A Borovac

**Affiliations:** 1 Cardiovascular Diseases Department, University Hospital of Split, 21000 Split, Croatia; 2 Department of Physiology, University of Split School of Medicine, 21000 Split, Croatia; 3 Department of Health Studies, University of Split, 21000 Split, Croatia; 4 Department of Pathophysiology, University of Split School of Medicine, 21000 Split, Croatia

**Keywords:** SGLT2 inhibitors, right ventricular function, two-dimensional speckle tracking echocardiography, three-dimensional echocardiography, remodeling, heart failure with reduced ejection fraction

## Abstract

**Background:** The impact of sodium-glucose cotransporter-2 inhibitors (SGLT2is) in addition to optimal medical therapy (OMT) on the right ventricular (RV) systolic function using advanced echocardiographic analysis among outpatients with heart failure and a reduced ejection fraction (HFrEF) has thus far been poorly investigated. **Methods:** This was a single-center, prospective, single-blinded study in which an echocardiographic expert was blinded to the allocation of the treatment. A total of 36 outpatients with HFrEF were randomized to either OMT or OMT+SGLT2i. Both groups underwent an echocardiographic examination of the RV systolic function at the baseline and at the 3-month follow-up (3mFU). **Results:** The patients in both groups did not significantly differ with respect to the relevant baseline comorbidities, therapy, and clinical characteristics. The patients receiving OMT+SGLT2i showed a significant improvement from the baseline to the 3mFU in all the measured RV echocardiographic parameters, while for the OMT group, a significant improvement after the 3mFU was observed for TAPSE and s’. The mean percent change from the baseline to the 3mFU was significant when comparing OMT+SGLT2i to the OMT group concerning RV FWS (+91% vs. +28%, *p* = 0.039), TR maxPG (−27% vs. +19%, *p* = 0.005), and TR Vmax (−17% vs. +13%, *p* = 0.008), respectively. **Conclusions:** Adding SGLT2i to OMT in patients with HFrEF resulted in a greater improvement in the RV systolic function from the baseline to the 3mFU compared to the OMT alone.

## 1. Introduction

Sodium-glucose co-transporter 2 inhibitors (SGLT2i) have emerged as a fundamental therapy for heart failure (HF) across the spectrum of ejection fractions. Recent results from the EMPEROR-Preserved and the DELIVER randomized trials have shown that SGLT2i (empagliflozin and dapagliflozin, respectively) can reduce all-cause mortality and hospitalizations due to HF in patients with HF with a mildly reduced or preserved ejection fraction [1,2]. The guidelines for the treatment of patients with heart failure with an reduced ejection fraction (HFrEF) by the European Society of Cardiology from September 2021 set SGLT2i as a pillar therapy with an IA class recommendation based on the results of the EMPEROR-Reduced and DAPA-HF trials [3,4,5].

The beneficial effects of SGLT2i on the left ventricular systolic and diastolic function, circulating natriuretic peptide levels, and functional symptom burden have been demonstrated in several studies, but not much is known about the effects of these drugs on the right ventricular (RV) systolic function [6,7]. The RV is often neglected because of its complex anatomy and the difficulty of obtaining satisfactory imaging “windows” in daily practice [8,9,10]. However, the RV has been shown to play an important prognostic role following cardiac surgery and in patients with HF, pulmonary arterial hypertension, or ischemic heart disease [11,12,13]. In clinical practice, the assessment of the RV function is usually undertaken by measuring only the longitudinal systolic function, as reflected in the measurement of the tricuspid annular plane systolic excursion (TAPSE) and tricuspid lateral annular systolic velocity (s’ wave) derived from Doppler tissue imaging. The fractional area change (FAC), on the other hand, gives us an insight into the radial contraction of the RV [8]. Because these parameters are load- and angle-dependent, advanced echocardiographic methods, such as speckle-tracking echocardiography (STE) measuring the longitudinal strain of the RV free wall (RV FWS) and the three-dimensional RV ejection fraction (3D RVEF), have recently emerged as more accurate estimates of the RV systolic function [8,11]. The measurement of the 3D RVEF overcomes the limitations of the geometric assumption of the RV and integrates both the longitudinal and radial components of the myocardial muscle contraction, whereas TAPSE and s’ wave measure only the longitudinal RV function in the basal region of the RV free wall [10]. The 3D echocardiography assessment of the RV ejection fraction and RV FWS have been shown to be comparable to the gold standard cardiac magnetic resonance (CMR) [11,14,15] and were shown to be an independent predictor of cardiac death and major adverse cardiovascular events (MACE) in patients with diverse cardiovascular diseases [14,16,17,18]. Furthermore, the ratio between the TAPSE and systolic pulmonary artery pressure (SPAP) represents the non-invasive measurement of the right ventriculo-arterial coupling and the RV contractile function [19].

For these reasons, we designed the present study to investigate the potential effects of SGLT2is on the RV systolic function after it was added to optimal medical therapy (OMT) for HFrEF. 

## 2. Materials and Methods

### 2.1. Study Design, Inclusion, and Exclusion Criteria

This was a single-center, prospective, single-blinded study that was conducted from March 2021 to September 2021 at the Cardiovascular Disease Department, the University Hospital of Split, Croatia, in full compliance with the principles of the Declaration of Helsinki from 2013 and with the approval of the Ethics Committee of the University Hospital of Split under number 2181-147/01/06/M.S.-20-02. All the participants read and signed the informed consent form. We consecutively enrolled 36 outpatients with HFrEF according to the European Society of Cardiology guidelines (ESC) that were endorsed at the time of the initiation of the study (2016 edition) [20].

The inclusion criteria were: that the patients were diagnosed with HFrEF according to the ESC guidelines for the diagnosis and management of acute and chronic heart failure [20] and that they were aged 18 years or older with verified LVEF < 40%. At the time of inclusion, the patients were already required to receive background optimal guideline-directed medical therapy (OMT) at the highest tolerated daily doses of medications, including sacubitril-valsartan (angiotensin receptor neprilysin inhibitor—ARNi), beta-blocker (BB), and the mineralocorticoid receptor antagonist (MRA), in addition to other symptomatic therapies such as loop diuretics. In addition, only patients with a functional symptom severity class II and III, as assessed by the New York Heart Association (NYHA) scale, and a concurrent N-terminal-pro-b-type natriuretic peptide (NT-proBNP) value >125 pg/mL were included in the study.

Patients were excluded if they had: symptomatic hypotension (systolic blood pressure < 95 mmHg), an impaired renal function (eGFR < 30 mL/min/1.73 m^2^ calculated according to the CKD-EPI formula) and serum potassium level > 5.2 mmol/L, hepatic dysfunction (defined as liver parameters such as ALT, AST, and/or ALP, which are three times above the upper 99-th percentile of the reference range, biliary cirrhosis and cholestasis, active malignancies (regardless of the stage and type of malignancy)), the current use of hormone replacement therapy, chemotherapy, or immunotherapy, the presence of an artificial heart valve (mechanical or biological), severe aortic stenosis, acute coronary syndrome in the three months preceding their enrollment in the study, percutaneous coronary intervention, or acute cerebrovascular incident in the last three months before the date of enrollment, pregnancy, or if they were breastfeeding. In addition, patients with diabetes mellitus treated with DPP4 inhibitors and GLP receptor agonists were excluded from the study because of possible interactions with the structure and function of the myocardial. Finally, patients who were unable to provide informed consent or declined to participate in the study were not enrolled.

After obtaining written informed consent, patients were consecutively randomized into two groups using a random number generator: (a) the OMT + SGLT2 inhibitor group (N = 18)—these patients received the SGLT2 inhibitor (either empagliflozin or dapagliflozin 10 mg once daily) in addition to background OMT—and (b) the OMT control group (N = 18); these patients received background OMT without the addition of SGLT2i. After their allocation to a therapy regimen, all patients were assigned to a standard and extended transthoracic echocardiographic (TTE) examination, physical examination, and biochemical laboratory testing at the baseline and the 3-month follow-up (3mFU).

Before the TTE examination, a detailed medical interview was performed regarding comorbidities, pharmacotherapy with daily doses for each treatment, their smoking status, and a physical examination with noninvasive measurements of taking their arterial blood pressure. The arterial blood pressure was determined as the mean of three consecutive measurements in the left antebrachial region. The functional NYHA status was determined in all patients. Peripheral venous blood samples for laboratory analyzes were collected on the morning of the examination day after overnight fasting and before the TTE examination. The samples were properly stored and analyzed by the same medical biochemistry specialist who was also blinded to the assignment of a treatment to the participants. All laboratory tests were performed according to good laboratory practice and included the measurement of the complete blood count (CBC), fasting plasma glucose, serum urea, creatinine, uric acid, electrolytes (sodium-Na, potassium-K), aspartate aminotransferase (AST), alanine, aminotransferase (ALT), gamma-glutamyl transferase (GGT), lactate dehydrogenase (LDH), C-reactive protein (CRP), and N-terminal-pro-brain natriuretic peptide (NT-proBNP) levels. The laboratory measurements were performed in all patients on the day of the first examination and repeated in the same fashion at the 3mFU.

### 2.2. Echocardiographic Examinations

All TTE examinations were performed with the patient at rest in the left supine position using the same commercially available ultrasound system (Vivid 9E, GE Medical System, Milwaukee, WI, USA). All the echocardiography data were digitally stored and analyzed on the Echo PAC workstation (Echo PAC 202 PC, GE Medical System, Milwaukee, USA). 

The right ventricular systolic function was assessed with advanced TTE methods: two-dimensional STE evaluating the RV longitudinal strain of the RV free wall (RV FWS, −%) and three-dimensional RV ejection fraction (3D RVEF, %) using 3D echocardiography. The standard TTE parameters of the RV systolic function, such as the TAPSE, s’ wave, and FAC, were also determined. The TAPSE measurement was performed in the apical 4-chamber view by directing the M-mode cursor to the lateral tricuspid annulus and was calculated as the total systolic displacement of the annular segment in millimeters [11]. From the same view, using the tissue Doppler (TD) cursor through the lateral tricuspid annulus in the basal segment of the RV free wall, the s’ wave was calculated (cm/s), using the highest velocity of the systolic waveform [11]. In addition, from the apical 4-chamber view, the end-diastolic area (EDA) and end-systolic area (ESA) were calculated and the FAC was measured using the formula [(EDA − ESA)/EDA × 100] to obtain the percentage of the fractional area change. RV FWS was acquired from an RV focused apical 4-chamber view with an acquisition rate of 60 to 80 frames per second (fps) [11]. Using the two-dimensional STE of the three segments (the basal, middle, and apical segments) of the RV free wall, the mean peak systolic strain was measured by averaging the segmental peak values automatically generated by the software. The value of the RV FWS is expressed as a negative percentage because it represents longitudinal shortening, which is a reduction in the calculated speckle distance [18,21]. The example of “before–after” the RV FWS measurement is shown in Supplemental Figure S1.

To calculate the 3D RVEF, images were acquired with the 3D echo probe in apical four-chamber view using a pyramidal scan with a high image acquisition and wide-angle mode. The image was acquired in a cardiac cycle with 9 wedge-shaped subvolumes synchronized with electrocardiography while the patient held his breath for 7–10 s. The 3D RVEF measurement was performed at the workstation using EchoPAC software, with RV boundaries on the endomyocardium manually delineated and the RV volumes and ejection fraction automatically measured by the software [10,14]. The example of the 3D RVEF “before–after” measurement is shown in Supplemental Figure S2.

A color Doppler was placed over the tricuspid valve in the RV focused apical four-chamber view and a tricuspid regurgitation jet was defined. Then, using the continuous wave Doppler, the maximum tricuspid regurgitation velocity (TR Vmax, m/s) and the TR maximum pressure gradient (TR maXPG, mmHg) were calculated by tracing the TR spectrum. The systolic pulmonary artery pressure (SPAP) was calculated as the sum of the pressure gradient measured from the TR Vmax using the modified Bernoulli equation (4× (TR Vmax)2) and the right atrial pressure (mmHg) estimated on the basis of the inferior vena cava size and collapsibility during inspiration [22]. The ratio between the calculated values of the TAPSE and SPAP was then measured (mm/mmHg).

### 2.3. Outcome Measures

The primary endpoint was defined as the change in the RV systolic echocardiographic measurements (RV FWS, RV 3DEF, TAPSE, s’ wave, FAC, TAPSE/SPAP, TR Vmax, and TR PPG) from the baseline to 3 months of continuous SGLT2i therapy combined with OMT in HFrEF patients.

The secondary endpoints were the differences in the mean percent change in the RV systolic echocardiographic measurement in patients receiving SGLT2i in addition to OMT compared with HfrEF patients receiving identical OMT without the addition of SGLT2i.

### 2.4. Sample Size Calculation

The sample size calculation and power analysis were conducted a priori by using the projected difference between the two independent means (the two groups of interest—OMT vs. OMT + SGLT2i). Left ventricular global longitudinal strain (LV GLS) is currently used in cardio-oncology as a tool to prevent cancer therapy-related cardiovascular toxicity and a significant change from the baseline to follow-up in patients receiving cancer-related therapy is defined as a 15% decrease in LV GLS from the baseline [23]. Because RV FWS has not been well studied, similar changes have not been described previously, but by analogy and considering the normal RV FWS value being greater than −19% [8], we defined a meaningful difference in the RV FWS between the two groups at the value of 2.0% ± 1.5%. These input parameters produced an effect size (d) of 1.33 with an alpha error (α) probability and statistical power (1- β error probability) defined at 0.05 and 0.95, respectively. Based on these assumptions, a total sample size of 26 patients was required for the study (*n* = 13 in each group).

### 2.5. Study Reliability

All the echocardiographic measurements were performed by the same cardiologist with a high level of expertise in echocardiography who was blinded to the allocation of a treatment to the participants. To reduce the intraobserver error, all the measurements from the beginning of the study were revised at the end of the study at the Echo PAC workstation. To reduce the interobserver error, the echocardiographic measurements were validated by another cardiology consultant with a high expertise in echocardiography to determine the possibility of a measurement error. The analysis of correlation and agreement between the expert echocardiographer assigned to the study and the control expert echocardiographer was performed by using Bland–Altman analysis and is available as Supplemental File S1.

### 2.6. Statistical Analysis

All the data analyses were performed by using SPSS Statistics for Windows, version 23.0 (IBM Corp., Armonk, NY, USA) and Prism, version 9.0.1. (Graphpad, La Jolla, CA, USA). Continuous data were, depending on the variable normality of distribution, shown as the mean ± standard deviation (SD) or median (interquartile range), while the categorical variables were displayed as whole numbers (N) and percentages (%). The normality of the data distribution was examined with the Kolmogorov–Smirnov test. The t-test for independent samples for variables with a normal distribution and Mann–Whitney U test for the variables with a non-normal distribution were utilized to measure the potential differences between the two groups of interest (OMT+SGLT2i vs. OMT group). The echocardiographic variables of interest were specifically examined in the “before–after” fashion in which the initial values (at the baseline) of each individual patient were pairwise compared to the values obtained at the follow-up. The mean absolute change (Δ, delta) in these values from the baseline to the 3mFU was reported for each group of interest and these values were compared between the groups by using a t-test. Similarly, for the purpose of a main analysis, the mean percent change from the baseline to the 3mFU was calculated for each group by using formula [(Measurement_2_ − Measurement_1_)/Measurement_1_] × 100 and these values were then compared between both groups by using an independent samples *t*-test. The Chi-squared (χ^2^) test was used to examine the differences between the groups of interest with respect to the categorical variables, also for the measurements of the proportion of patients from each group that reached the cut-off value of RV FWS, which was 16% and above. Two-tailed significance values (*p*) were reported in all instances, while the results that reached *p* < 0.05 were considered to be statistically significant.

## 3. Results

### 3.1. Patients’ Baseline Characteristics

A total of 36 consecutive HfrEF outpatients were randomized in the study. The baseline characteristics of the patients randomized to OMT+SLGT2i (*n* = 18) vs. OMT alone (*n* = 18) did not significantly differ concerning the age, sex, NYHA functional class, renal function, etiology of cardiomyopathy, relevant comorbidities (arterial hypertension, diabetes mellitus, dyslipidemia, and atrial fibrillation), and mean daily dose or distribution of the chronic HF-related therapies, as it can be appreciated from Table 1. Importantly, all the patients in both groups received the identical background OMT consisting of ARNi, BB, and MRA.

Furthermore, both groups did not significantly differ with respect to the baseline laboratory indices while they were similar in the baseline echocardiographic parameters reflecting the left ventricular systolic and diastolic function (Table 1).

In both of the examined groups, the echocardiographic parameters of the right ventricular systolic function were predominantly reduced, however, the obtained values measured at the baseline echocardiographic examination were similar in both groups (Table 2).

### 3.2. Absolute Mean Changes in Advanced Echocardiographic Parameters of Right Ventricular Function in Each Treatment Group, from Baseline to 3mFU

Patients with HfrEF randomized to the OMT+SGLT2i treatment experienced a significant improvement from the baseline to the 3mFU in all the RV functional echocardiographic parameters that were measured, as reflected in the mean absolute change, as follows: TAPSE (+4.5 mm, *p* = 0.002), s’ (+3.5 cm/s, *p* = 0.032), 3D RVEF (+10.1%, *p* = 0.003), RV FWS (+7.2%, *p* < 0.001), and RV FAC (+9.0%, *p* = 0.029). On the other hand, the improvement from the baseline to the 3mFU in the OMT-only group was significant only for the TAPSE and s’ wave (+2.4 mm, *p* = 0.040 and +2.7 cm/s, *p* = 0.013, respectively), while the 3D RVEF, RV FWS, and RV FAC were all associated with a numerical improvement but failed to reach a statistical significance (Table 3).

The echocardiographic parameters measuring the tricuspid regurgitation maximal velocity (TR V_max_) and maximum pressure gradient (TR maxPG) were significantly reduced from the baseline to the 3mFU in patients that were randomized to the OMT+SGLT2i treatment (−0.7 m/s, *p* = 0.003 and −11.5 mmHg, *p* = 0.002, respectively). Contrary to this, no significant changes regarding the tricuspid regurgitation echocardiographic parameters were observed in the patients randomized only to OMT (Table 3). The TAPSE/SPAP ratio did not differ between the groups at the baseline (Table 2), and both groups showed no improvement after the 3mFU with a greater improvement in the OMT+SGLT2i group compared with the OMT-control group but without a statistical significance (Table 3). 

### 3.3. Mean Percent Change in Echocardiographic Parameters of RV Systolic Function between OMT+SGLT2i and OMT Groups, from Baseline to 3mFU 

**Figure 1:**
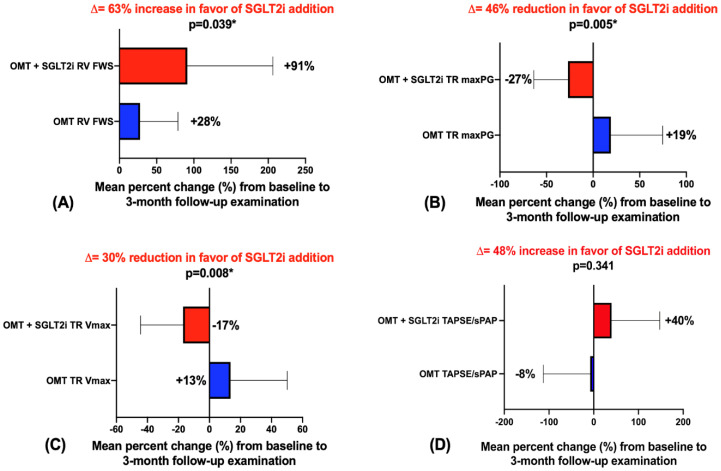
Mean percent change (%) from baseline to 3-month follow-up; red—OMT+SGLT2i group, blue—OMT control group; (**A**)—mean percent change in right ventricular free wall strain between groups from baseline to 3mFU; (**B**)—mean percent change in TR maximal pressure gradient between groups from baseline to 3mFU; (**C**)—mean percent change in maximal TR velocity between groups from baseline to 3mFU; (**D**)—mean percent change in TAPSE/SPAP ratio between groups from baseline to 3mFU. * Two-tailed significance values (*p*) were reported in all instances, while the results that reached *p* < 0.05 were considered to be statistically significant.

The analysis directly comparing the mean percent change (%) from the baseline to the 3mFU demonstrated a greater numerical improvement for all the RV systolic function parameters in the OMT+SGLT2i vs. OMT group, however, a statistical significance was reached only for RV FWS, TR V_max_, and TR maxPG (Figure 1). It is of note that the addition of SGLT2i to OMT was associated with a mean 63% increase in the RV FWS compared to the OMT-only group (Figure 1A), while a tricuspid regurgitation was significantly more reduced in the OMT+SGLT2i group compared to the OMT-only group (46% reduction in TR maxPG and 30% reduction in TR V_max_, Figure 1B and 1C, respectively).

The TAPSE/SPAP ratio showed a mean percent increase of 48% from the baseline to the 3mFU in favor of the OMT+SGLT2i+ group compared with the OMT-group, but did not reach a statistical significance when comparing between the groups (Figure 1D). 

**Figure 2:**
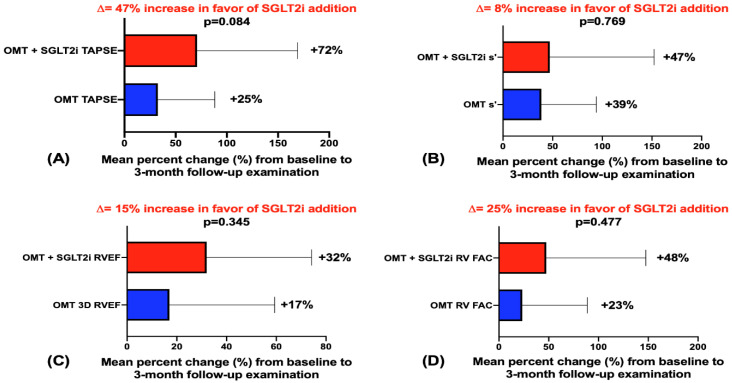
Mean percent change (%) from baseline to 3-month follow-up; red—OMT+SGLT2i group, blue—OMT control group; (**A**)—mean percent change in tricuspid annular plane systolic excursion (TAPSE) between groups from baseline to 3mFU; (**B**)—mean percent change in Doppler tissue imaging-derived tricuspid lateral annular systolic velocity (s’ wave) between groups from baseline to 3mFU; (**C**)—mean percent change in right ventricular ejection fraction measured by three-dimensional echocardiography between groups from baseline to 3mFU; (**D**)—mean percent change in fractional area change (FAC) between groups from baseline to 3mFU.

Furthermore, TAPSE showed 47% (*p =* 0.084) a greater improvement from the baseline to the 3mFU in favor of the OMT+SGLT2i treatment, the s’ wave increased by 8% (*p =* 0.769) in the OMT+SGLT2i group, the FAC increased by the mean of 25% (*p =* 0.477) in the OMT+SGLT2i group, and the 3D RVEF increased by 15% more in the OMT+SGLT2i (*p =* 0.345) compared to the OMT-only group (Figure 2A, 2B, 2C, and 2D, respectively). 

When combined together and averaged, the global improvement in the RV hemodynamics encompassing all the measured RV systolic parameters yielded an average 33 ± 10% improvement in the OMT+SGLT2i group compared to the OMT-only group, from the baseline to the 3 mFU (*p* = 0.006, Supplemental Figure S3).

**Figure 3:**
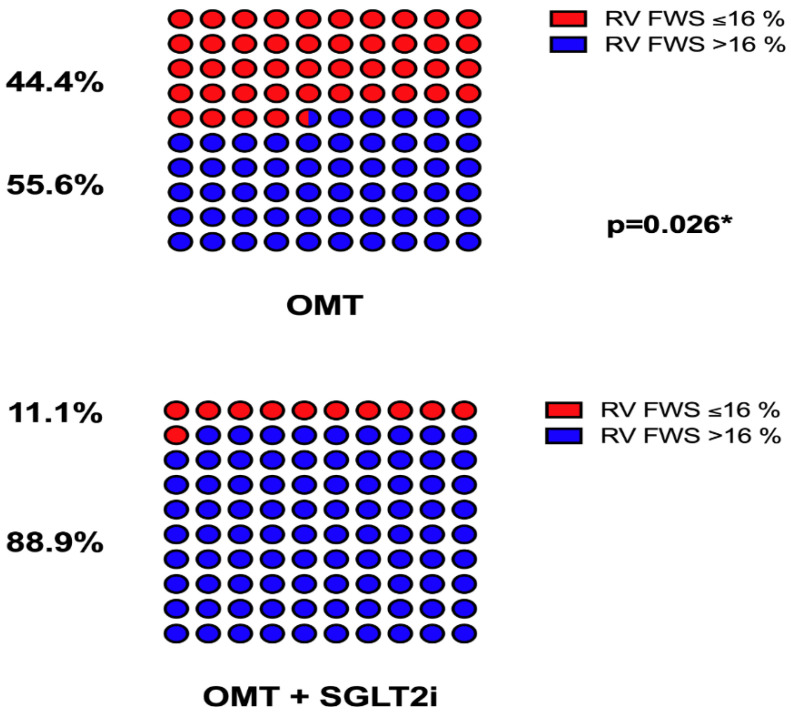
Upper image—proportion of patients from the OMT control group with the right ventricular free wall strain value (RV FWS) of −16% or more positive to absolute zero value (red color); or above −16% and more negative value from absolute zero value. Bottom image—proportion of patients from the OMT+ SGLT2i+ group with the right ventricular free wall strain value (RV FWS) of −16% or more positive to absolute zero (red color); or over −16% and more negative value from absolute zero.

Finally, as demonstrated in Figure 3, a group of patients that had SGLT2i added to OMT had a significantly lesser proportion of impaired RV FWS (≤16%) at the 3 mFU, compared to patients that received OMT only (11.1% vs. 44.4%, *p* = 0.026).

It should also be noted that the echocardiographic measurements at the baseline and the 3mFU visit showed a low degree of discrepancy between the expert sonographer responsible for all the measurements undertaken in the study and the control expert sonographer (results in Supplemental File S1).

## 4. Discussion

In this prospective study in which consecutive outpatients with HFrEF were randomly allocated to a treatment group, we demonstrated an echocardiographic improvement in the RV systolic function from the baseline to the follow-up visit at 3 months among the patients that had SGLT2i added to OMT compared to those that received OMT only. Furthermore, the addition of SGLT2i to OMT appeared to significantly reduce the degree of tricuspid regurgitation compared to a treatment with OMT alone. 

The exact effects of SGLT2i on the RV function have not been thus far studied in great detail. Patoulias and colleagues emphasized the need to evaluate the effects of SGLT2i on the RV function, citing the RV as a “forgotten” cardiac chamber with significant knowledge gaps [7]. A post hoc analysis of the EMPA-HEART CardioLink-6 trial in patients with type II diabetes mellitus and coronary artery disease (CAD) showed no differences in the RV mass index, RV volume, and RV EF, measured by CMR after 6 months of empagliflozin compared with placebo [24]. Conversely to this, a recent retrospective study demonstrated a significant improvement in the pulmonary artery’s stiffness and RV systolic function in HFrEF patients after 6 months of SGLT2i therapy compared to the baseline, as measured by TAPSE, s’ wave, and FAC, along with a significant decrease in the mean pulmonary systolic pressure [25]. However, to the best of our knowledge, our study is the first one that prospectively examined the effects of an SGLT2i addition to OMT in HFrEF outpatients on the RV systolic function using advanced 3D echocardiography and 2D speckle-tracking of the RV free wall.

It is important to emphasize that our results were obtained in groups that were well matched on many relevant baseline covariates. All of the patients in both groups received the same OMT, which was maximally up-titrated to the individual patient and consisted of foundational HFrEF therapy with ARNi, BB, MRA, and furosemide. There were no differences between the groups in the average daily dose of the therapies. No therapy was titrated or suspended during the follow-up period because the participating patients had already received the maximum tolerated therapy. More importantly, the included HFrEF population did not differ in either the systolic or diastolic left ventricular function and had similar values of LV GLS and LV 3DEF. 

Mouton and colleagues emphasized a cut-off value of RV FWS < −16% for the diagnosis of the RV systolic dysfunction with a high specificity and moderate sensitivity for poor outcomes in the HFrEF population [26]. Our results showed that 4 times fewer patients in the OMT+SGLT2i group had RV FWS ≤ −16% than patients only receiving OMT alone. Moreover, RV FWS is not only a prognostic parameter but is also able to detect the subtle deterioration of the RV systolic function despite the preserved TAPSE, s’ wave, and FAC in HF patients [27,28]. Another important feature of RV FWS is that it likely reflects the extent of RV myocardial fibrosis in the later stages of HFrEF development [29]. These observations are important in the context of our results, as our study showed a numerical improvement in all the measured parameters of the RV systolic function from the baseline to the 3mFU in patients receiving SGLT2i in addition to OMT, however, RV FWS was the only echocardiographic indicator that was statistically significant improved compared to the OMT-only group.

Thus far, studies on the impact of ARNi in HFrEF patients showed an improvement in left and right ventricular function [30,31]. According to a recent meta-analysis, ARNi improves the right ventricular function and reduces the pulmonary hypertension independent of the left ventricular reverse remodeling [32,33,34]. In our study, all the enrolled patients were taking ARNi as the background therapy in maximally uptitrated doses that were tolerated by the patients. The group of patients not receiving SGLT2i after 3 months of a follow-up showed a numerical improvement in all the measured RV parameters, but only the TAPSE and s’ wave reached a statistical significance, which is in accordance with previous studies [31]. 

The results of the EMBRACE-HF study focused on HF patients in a wide spectrum of LVEF and regardless of the DM status with NYHA class III-IV and the pulmonary arterial hypertension (mean diastolic pulmonary artery pressure(PAP) of 22 mmHg) showed that PAP was reduced with empagliflozin compared with the placebo after 12 weeks of therapy, but the effects on the RV were not studied [35]. Previously, studies using animal models have shown a reduction in the mean PAP under SGLT2i therapy and a reduction in the RV hypertrophy [24,36,37]. The results of our study clearly show the same signal, as there was a highly statistically significant reduction in the TR Vmax and TR maxPG from the baseline to the 3mFU in patients receiving SGLT2i in addition to maximally uptitrated OMT, whereas these parameters remained similar after the 3mFU in patients receiving maximally uptitrated OMT alone. Importantly, a significantly greater reduction in the tricuspid regurgitation parameters was retained when OMT+SGLT2i and OMT-only groups were directly compared. It should be emphasized that patients in both groups had a similar degree of RV systolic dysfunction, mild tricuspid regurgitation, and a low probability of pulmonary hypertension calculated by TAPSE/SPAP at the baseline [38]. The estimate of TAPSE/SPAP has a high predictive value and correlates with the stiffness of the pulmonary artery [39,40], which is important given our results of a greater improvement from the baseline to the 3mFU in patients receiving SGLT2i comparing to OMT-only patients. 

The exact pathophysiological mechanisms explaining these results waits to be elucidated. However, the beneficial role of SGLT2i in reducing the extent of pulmonary hypertension and RV remodeling can be explained by their multifactorial and pleiotropic effects. SGLT2i have metabolic, vascular, and hemodynamic effects. They reduce body weight due to a renal caloric loss by glycosuria, have beneficial effects on the cardiac metabolism, and improve the cardiac energetics [41]. They also reduce myocardial oxidative stress, and by inhibiting the myocardial sodium-hydrogen exchanger 1 (NHE1), they the reduce cytoplasmic sodium and calcium levels [41,42]. The combination of the different mechanisms prevents cardiac remodeling. Due to the mechanism of osmotic diuresis, the initial volume depletion results in a decrease in the pulmonary pressure within the first few days after the initiation of the treatment [35]. The patients in our study did not differ in terms of the average diuretic dose, so the possible explanation for the SGLT2i effect is the addition of the osmotic diuretic and natriuretic effects, which led to a reduction in the RV preload. In addition, SGLT2 inhibitors attenuate the activation of the renin-angiotensin-aldosterone system (RAAS) and reduce the discharge of the sympathetic nervous system, which in turn attenuates systemic and pulmonary arterial stiffening [34,35,43]. Camci and Yilmaz demonstrated the beneficial role of SGLT2i in reducing the pulmonary arterial stiffness (PAS) wherefore patients exhibit a better pulmonary vascular compliance, which attenuates the RV afterload and thus improves the RV systolic function [25]. Additionally, the previously demonstrated reduction in the LV filling pressure and improvement in the LV diastolic function is reflected in the improvement in the RV function [6,44]. Another beneficial effect of SGLT2i that may explain the improvement in the RV is its action on vascular cells through an anti-inflammatory and antioxidant effect, also increasing the angiogenesis and nitric oxide bioavailability from the endothelium, leading to pulmonary and systemic vasodilation, thereby reducing the RV preload and afterload [45,46].

There are some limitations to our work that should be noted. This was a single-center study enrolling an ethnically homogeneous population, thus these observations might not be able to be generalized to the overall or worldwide population. Furthermore, given that this was a “concept-generating” study that enrolled a rather small number of HFrEF patients, it is possible that some of the presented results would reach a statistical significance if a larger number of patients was enrolled. It is also possible that a timeframe greater than 3 months and a longer follow-up might be required to show a greater benefit of the addition of SGLT2i to background OMT for the improvement in the RV function in HFrEF. Similarly, a low number of patients might have generated random differences that could impact on the obtained results. Our study was not powered nor designed to capture clinical outcomes such as death and/or hospitalization events, although we can state that all patients were well-compensated at their second examination and none of them died or required hospitalization within 1 year from the follow-up examination based on our follow-up records. However, the strengths of this study are that both of the compared groups were well-matched in important baseline covariates and were treated with the same background OMT that was, correspondingly, nearly identical with respect to the daily doses. Finally, the patients were consecutively enrolled and randomly allocated a treatment, while all the measurements were recorded by the single sonographer with a high expertise in advanced echocardiography who was blinded to the allocation of the treatment. Last but not least, the echocardiographic measurements were validated against another expert sonographer.

## 5. Conclusions

This concept-generating study demonstrated that among outpatients with HFrEF, the addition of SGLT2i to maximally uptitrated OMT resulted in a significant improvement in the RV systolic function from the baseline to the 3mFU. Moreover, a significant improvement among the OMT+SGLT2i vs. OMT-only patients was demonstrated in the parameters of the RV free wall strain and the parameters reflecting the degree of tricuspid regurgitation. Taken together, our results suggest that the addition of SGLT2i to background OMT provides an incremental benefit concerning the RV hemodynamics in outpatients with HFrEF. Further large-scale studies are welcome and warranted to confirm these initial findings.

## Figures and Tables

**Table 1:** Baseline characteristics of two treatment groups (OMT vs. OMT + SGLT2 inhibitor).

Variable	OMT (*n* = 18)	OMT + SGLT2i (*n* = 18)	*p*-Value *
Age, years	67.8 ± 12.7	67.3 ± 11.5	0.902
Male sex, %	16 (88.9)	16 (88.9)	1.000
NYHA functional class	2.2 ± 0.38	2.3 ± 0.49	0.371
Smoking, %	6 (33.3)	8 (44.4)	0.494
Dyslipidemia, %	11 (61.1)	10 (55.6)	0.735
Arterial hypertension, %	13 (72.2)	14 (77.8)	0.700
Diabetes mellitus, %	4 (22.2)	7 (38.9)	0.154
Atrial fibrillation, %	7 (38.8)	5 (27.8)	0.297
Ischemic cardiomyopathy, %	9 (50.0)	6 (33.3)	0.310
Systolic blood pressure, mmHg	123 ± 18	124 ± 19	0.860
Diastolic blood pressure, mmHg	73 ± 10	74 ± 14	0.782
Heart rate, bpm	81 ± 21	80 ± 25	0.965
3D LVEF, %	28.5 ± 8.5	30.2 ± 9.7	0.573
LV GLS, %	−7.8 ± 3.8	−7.8 ± 3.7	0.965
LVEDV, mL	223 ± 71	236 ± 85	0.609
LVESV, mL	139 ± 67	145 ± 72	0.782
LA volume, mL	81 ± 36	81 ± 27	0.988
E/E’	13.6 ± 5.2	13.8 ± 5.5	0.901
MV E/A	1.21 ± 0.7	1.12 ± 0.5	0.647
AV V_max_, m/s	1.18 ± 0.35	1.17 ± 0.31	0.968
PVAT, msec	122 ± 32	120 ± 30	0.907
Hemoglobin, g/L	146 ± 15	144 ± 14	0.632
Fasting glucose, mmol/L	6.2 ± 2.1	6.8 ± 1.6	0.377
eGFR, mL/min./1.73 m^2^	64 ± 19	70 ± 17	0.295
Sodium, mmol/L	139 ± 2.0	138 ± 3.1	0.802
Potassium, mmol/L	4.4 ± 0.5	4.5 ± 0.8	0.772
C-reactive protein, mg/L	4.2 (0.9–7.2)	2.4 (1.0–6.9)	0.743
NT-proBNP, pg/mL	2326 (1188–5191)	3250 (1463–6227)	0.606
AST, IU/L	28 (25–36)	27 (23–33)	0.323
ALT, IU/L	39 ± 26	35 ± 14	0.544
GGT, IU/L	49 ± 32	67 ± 49	0.202
LDH, IU/L	191 (180–243)	206 (180–236)	0.864
ARNi + BB + MRA at baseline, %	18 (100)	18 (100)	1.000
Furosemide use, %	10 (55.6)	10 (55.6)	1.000
Statin use, %	10 (55.6)	10 (55.6)	1.000
Oral anticoagulant use, %	9 (50.0)	9 (50.0)	1.000
Sacubril-valsartan daily dose, mg	250 ± 115	239 ± 110	0.768
Beta-blocker daily dose, mg	3.75 ± 1.9	3.61 ± 1.3	0.803
MRA daily dose, mg	34.7 ± 12.5	33.3 ± 12.1	0.738
Furosemide daily dose, mg	61.4 ± 78.8	57.0 ± 91.3	0.886

Abbreviations: AV V_max_—aortic valve peak velocity; ALT—alanine aminotransferase; ARNi—angiotensin receptor neprilysin inhibitor; AST—aspartate aminostransferase; BB—beta blocker; E/A—peak velocity blood flow in early diastole to peak velocity blood flow in late diastole ratio; E/E’—early mitral inflow velocity to mitral annular early diastolic velocity ratio; eGFR—estimated glomerular filtration rate calculated by CKD-EPI formula; GGT—gama-glutamyl transferase; GLS-global longitudinal strain; LA—left atrium; LDH—lactate dehydrogenase; LVEDV-left ventricular end-diastolic volume; LVESV—left ventricular end-systolic volume; LVEF—left ventricular ejection fraction; MRA—mineralocorticoid receptor antagonist; NT-proBNP—N-terminal pro brain natriuretic peptide; PVAT—pulmonary velocity acceleration time; NYHA—New York Heart Association; * Results are presented as n (percent) and analyzed through Chi-squared test, mean ± standard deviation (t-test of independent samples) or median (interquartile range) analyzed through Mann–Whitney U test, based on variable normality.

**Table 2:** Right-sided functional echocardiographic parameters at baseline, stratified by the type of treatment received (OMT vs. OMT + SGLT2 inhibitor).

Variable	OMT (*n* = 18)	OMT + SGLT2i (*n*= 18)	*p*-Value *
TAPSE, mm	10.4 ± 3.7	9.2 ± 3.4	0.404
s’, cm/s	9.7 ± 4.5	11.1 ± 5.5	0.447
3D RVEF, %	38.8 ± 10.2	38.6 ± 8.5	0.936
RV FWS, %	−15.2 ± 5.6	−17.2 ± 6.3	0.488
FAC, %	34 ± 13	37 ± 14	0.462
TR maxPG, mmHg	22.7 ± 16.7	29.0 ± 19.2	0.290
TR V_max_, m/s	2.2 ± 0.9	2.6 ± 1.0	0.287
TAPSE/SPAP, mm/mHg	0.76 ± 0.70	0.76 ± 1.02	0.977

Abbreviations: FAC—fractional area change; RV—right ventricle; RV FWS—right ventricular free wall strain; TAPSE—tricuspid annular plane systolic excursion; TAPSE/SPAP—tricuspid annular plane systolic excursion/systolic pulmonary artery pressure; s′-tissue Doppler velocity of the basal free lateral wall of the right ventricle; TR maxPG—tricuspid regurgitant maximum pressure gradient; TR V_max_—tricuspid regurgitation max jet velocity; 3D RVEF—3D right ventricular ejection fraction. * Results are presented as mean ± standard deviation (t-test of independent samples).

**Table 3:** Absolute changes in values of right-sided functional echocardiographic parameters at baseline and 3-month follow-up, stratified by the type of treatment received (OMT vs. OMT + SGLT2 inhibitor).

Variable	OMT (*n* = 18)				OMT + SGLT2i (*n* = 18)			
	Baseline	3-Month Follow-Up	Δ Change Absolute	*p*-Value	Baseline	3-Month Follow-Up	Δ Change Absolute	*p*-Value
TAPSE, mm	10.4 ± 3.7	12.8 ± 5.0	+2.4	0.040 *	9.2 ± 3.4	13.7 ± 3.5	+4.5	0.002 *
s’, cm/s	9.7 ± 4.5	12.4 ± 5.4	+2.7	0.013 *	11.1 ± 5.5	14.6 ± 5.0	+3.5	0.032 *
3D RVEF, %	38.8 ± 10.2	42.3 ± 10.3	+3.5	0.432	38.6 ± 8.5	48.7 ± 9.8	+10.1	0.003 *
RV FWS, %	−15.2 ± 5.6	−18.5 ± 6.7	+3.3	0.067	−17.2 ± 6.3	−24.4 ± 5.8	+7.2	<0.001 *
RV FAC, %	34 ± 13	35.0 ± 10.8	+1.8	0.686	37 ± 14	46 ± 9	+9.0	0.029 *
TR maxPG, mmHg	22.7 ± 16.7	24.2 ± 16.0	+1.5	0.679	29.0 ± 19.2	17.6 ± 12.3	−11.5	0.002 *
TR V_max_, m/s	2.2 ± 0.9	2.5 ± 1.4	+0.3	0.248	2.6 ± 1.0	1.9 ± 0.7	−0.7	0.003 *
TAPSE/SPAP, mm/mmHg	0.76 ± 0.70	0.92 ± 1.04	+0.16	0.605	0.76 ± 1.02	1.39 ± 1.07	+0.63	0.079

Abbreviations: RV FAC—right ventricular fractional area change; RV—right ventricle; RV FWS—right ventricular free wall strain; TAPSE—tricuspid annular plane systolic excursion; TAPSE/SPAP—tricuspid annular plane systolic excursion/systolic pulmonary artery pressure; s′—tissue Doppler velocity of the basal free lateral wall of the right ventricle; TR maxPG—tricuspid regurgitant maximum pressure gradient; TR V_max_—tricuspid regurgitation max jet velocity; 3D RVEF—3D right ventricular ejection fraction. * Two-tailed significance values (*p*) were reported in all instances, while the results that reached *p* < 0.05 were considered to be statistically significant.

## Data Availability

Data sets generated and/or analyzed during the current study are available on reasonable request from the corresponding author.

## Supplementary Materials

The following supporting information can be downloaded at: https://www.mdpi.com/article/10.3390/jcm12010042/s1, File S1: The analysis of correlation and agreement between the expert echocardiographer assigned to the study and the control expert echocardiographer performed by using Bland–Altman analysis. Figure S1: The advanced echocardiographic measurement of right ventricular free wall strain at the (a) baseline and (b) 3-month follow-up examination; Figure S2: The advanced echocardiographic measurement of right ventricular 3D ejection fraction and other structural/functional parameters at the (a) baseline and (b) 3-month follow-up examination; Figure S3: Mean percent change (%) improvement in RV hemodynamics, encompassing all measured RV systolic parameters, from the baseline to 3mFU between the two groups.; red—OMT+SGLT2i group, blue—OMT control group.
