# Supplementary material for: Impact of SGLT2 Inhibitor Therapy on Right Ventricular Function in Patients with Heart Failure and Reduced Ejection Fraction
Source: J Clin Med. 2022 Dec 21;12(1):42. doi: 10.3390/jcm12010042 (PMC9820989; doi:10.3390/jcm12010042)

SUPPLEMENTAL FILE A

A BLAND-ALTMAN ANALYSIS OF CORRELATION AND AGREEMENT BETWEEN STUDY ECHO EXPERT *VS.* CONTROL ECHO EXPERT

Variable: Right ventricular free wall strain (%)

1st examination – 6 subjects
Average bias in measurement: 2.76 %
Average bias in absolute value (% RV FWS): 0.35 %
Standard deviation of bias: 5.82
95% Limits of agreement: -8.65 to 14.17

RV FWS at 1st examination
% Difference vs. average: Bland-Altman analysis

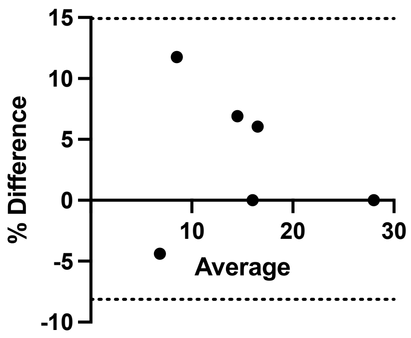

Follow-up examination – 6 subjects
Average bias in measurement: 4.97 %
Average bias in absolute value (% RV FWS): 1.38 %
Standard deviation of bias: 6.25
95% Limits of agreement: -7.28 to 17.21

RV FWS at follow-up examination
% Difference vs. average: Bland-Altman analysis

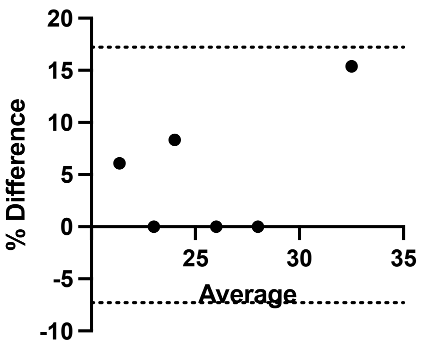

Variable: 3D right ventricular ejection fraction (%)

1st examination – 5 subjects
Average bias in measurement: 2.95 %
Average bias in absolute value (% RV FWS): 1.78 %
Standard deviation of bias: 12.67
95% Limits of agreement: -21.89 to 27.80

3D RVEF at 1st examination
% Difference vs. average: Bland-Altman analysis

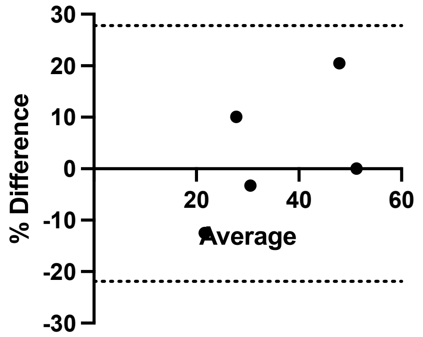

Follow-up examination – 6 subjects
Average bias: 0.56 %
Average bias in absolute value (% RV FWS): 0.27 %
Standard deviation of bias: 3.78
95% Limits of agreement: -6.86 to 7.97

3D RVEF at follow-up examination
% Difference vs. average: Bland-Altman analysis

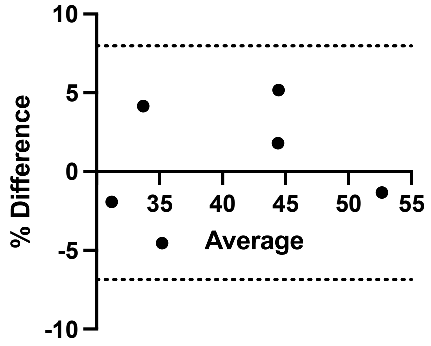

Supplement: Supplementary file 1 [file jcm-12-00042-s001.zip › File S1.pdf]
