# Supplementary figures and images for: Impact of SGLT2 Inhibitor Therapy on Right Ventricular Function in Patients with Heart Failure and Reduced Ejection Fraction
Source: J Clin Med. 2022 Dec 21;12(1):42. doi: 10.3390/jcm12010042 (PMC9820989; doi:10.3390/jcm12010042)

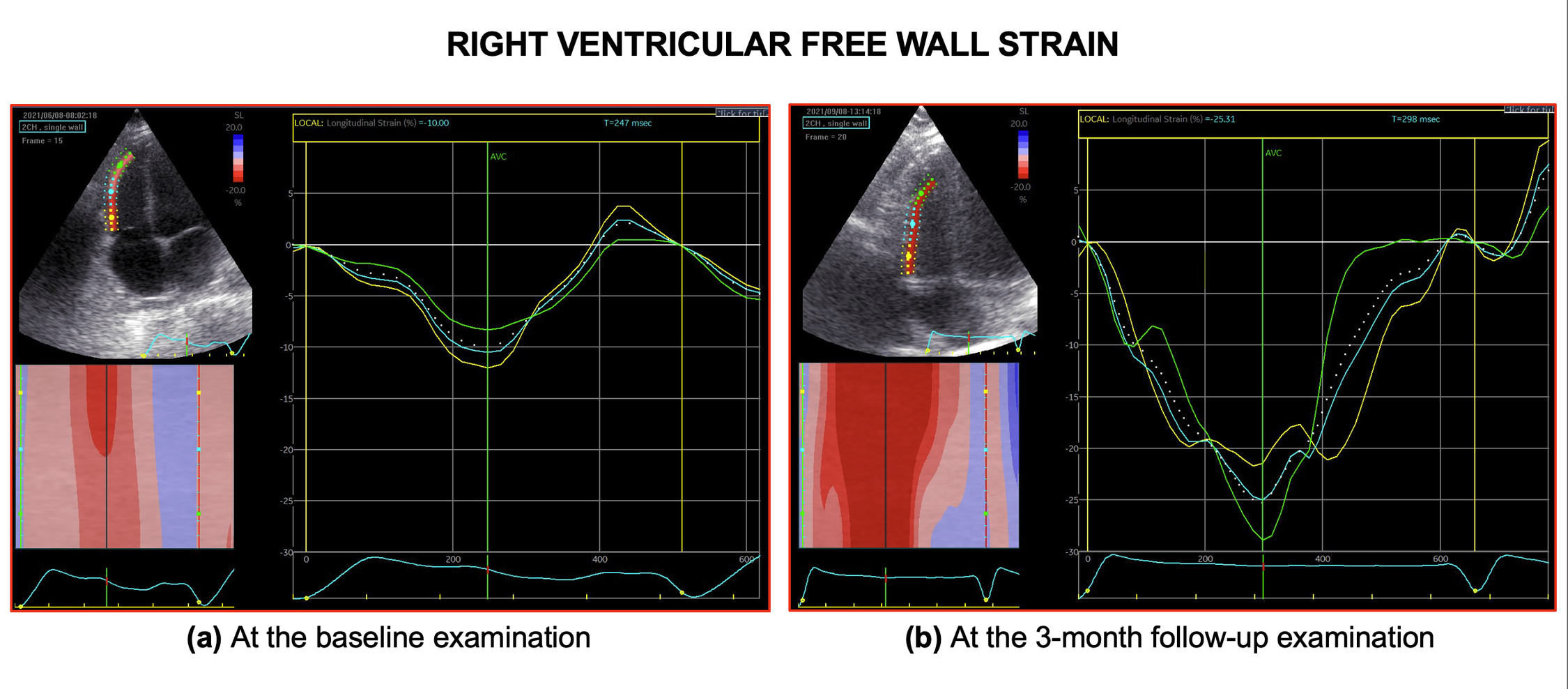

Supplement: Supplementary file 1 [file jcm-12-00042-s001.zip › Figure S1.tif]

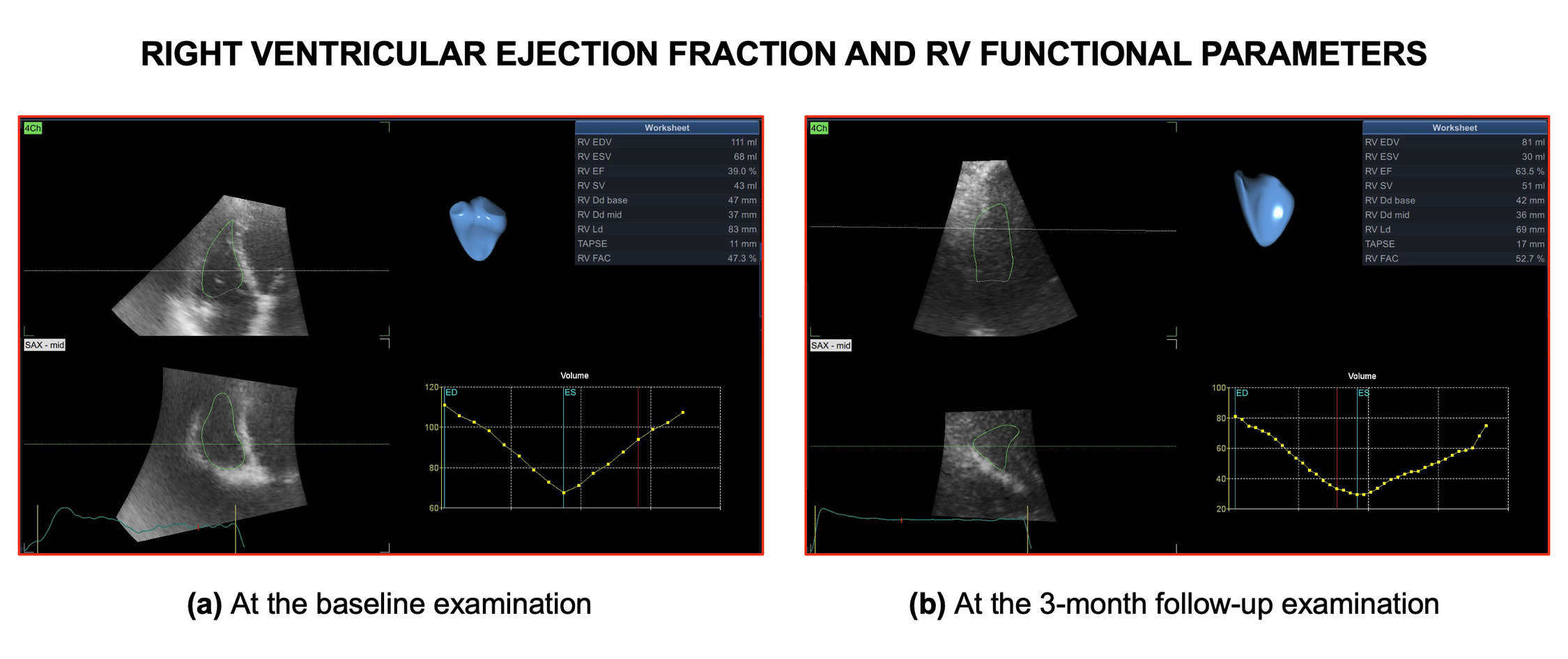

Supplement: Supplementary file 1 [file jcm-12-00042-s001.zip › Figure S2.tif]

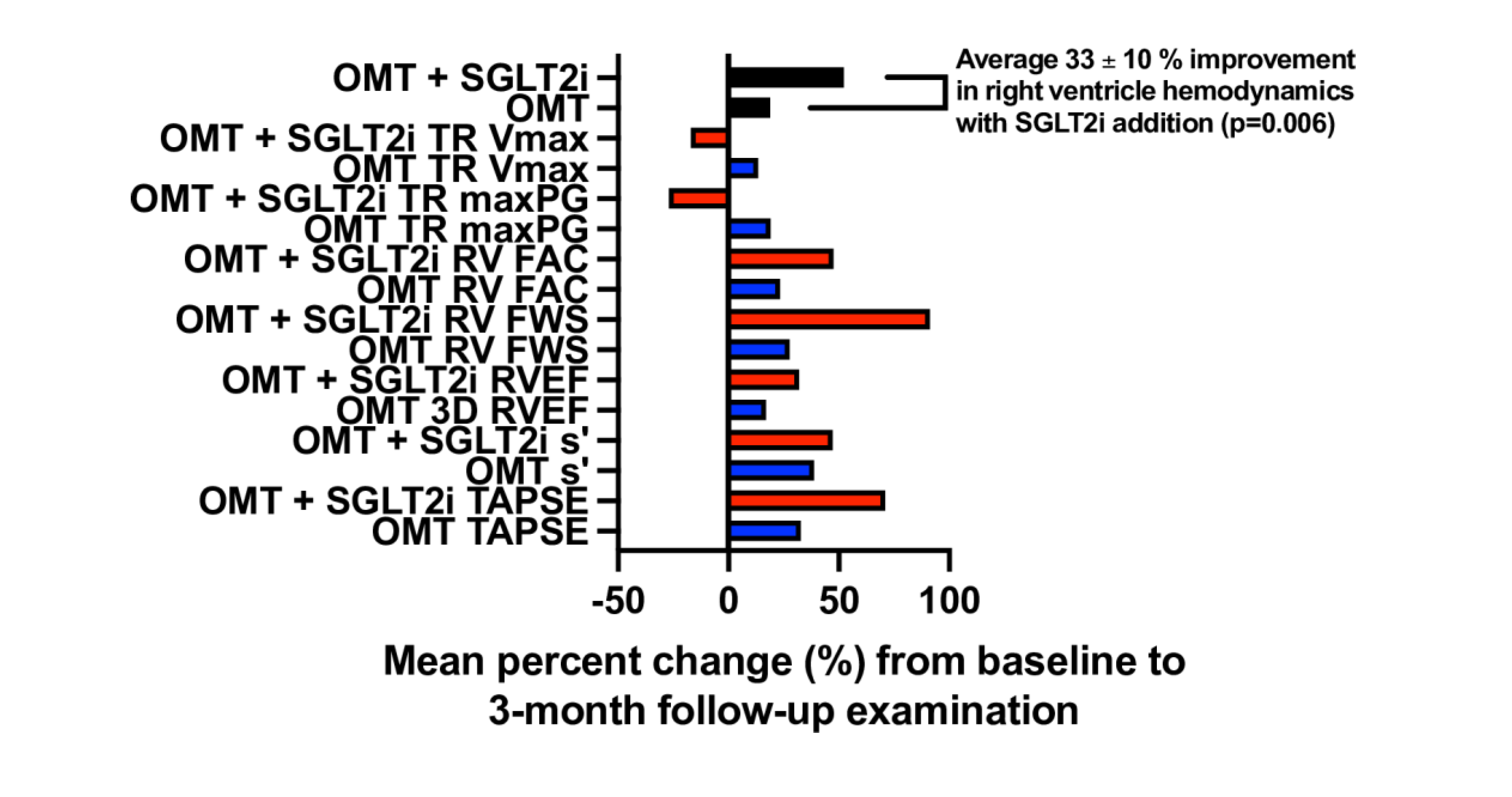

Supplement: Supplementary file 1 [file jcm-12-00042-s001.zip › Figure S3.tif]
